# Defatted biomass of the green microalga *Chlorella* sp. as a sustainable biostimulant to enhance barley growth under saline conditions

**DOI:** 10.1038/s41598-026-49609-6

**Published:** 2026-05-13

**Authors:** Ibrahim El-Akhdar, Tamer Elsakhawy, Ammar Elakhdar

**Affiliations:** 1 Agricultural Microbiology Research Department, Soils, Water and Environment Research Institute, Agricultural Research Center, Giza, 12112 Egypt; 2 Field Crops Research Institute , Agricultural Research Center, Giza, 12619 Egypt

**Keywords:** Salinity stress, Algal extract, Barley, Photosynthetic efficiency, Macronutrient accumulation, Plant sciences, Photosynthesis, Plant development, Plant reproduction, Plant stress responses

## Abstract

Defatted microalgal biomass represents a potentially sustainable resource for biostimulant production, reducing waste from biodiesel processing while enhancing crop resilience to abiotic stress. This study evaluated the potential of defatted *Chlorella* sp. extract as a biostimulant to alleviate salinity-induced stress in barley (*Hordeum vulgare*) grown hydroponically under electrical conductivity levels of 2, 8, and 12 mS cm⁻¹. Gas chromatography–mass spectrometry (GC–MS) analysis of the ethanol extract revealed 28 bioactive compounds, mainly fatty acids and esters associated with plant growth promotion and stress tolerance. Among the three barley genotypes tested (Giza 123, Giza 132, and Giza 134), Giza 123 exhibited the greatest resilience to salinity, showing the highest grain yield and photosynthetic activity. Application of algal extract, particularly through the combined seed-soaking and foliar spray treatment, led to marked improvements, increasing grain dry weight by 37%, electron transport rate (ETR) by 22%, and P, K, and N grain contents by 9–14% compared to the control. These enhancements were accompanied by higher protein content (9.6%) and chlorophyll a concentration (0.41 mg g⁻¹ FW). Significant genotype × treatment interactions indicated variable responses among cultivars. Overall, the results demonstrate that defatted *Chlorella* biomass extract serves as an effective and eco-friendly biostimulant, improving barley growth, nutrient uptake, and salinity tolerance while contributing to the circular valorization of algal residues.

**Supplementary Information:**

The online version contains supplementary material available at 10.1038/s41598-026-49609-6.

## Introduction

Aquatic ecosystems, despite their expanse, remain underexplored reservoirs of biologically active compounds^1^. Algae distinguish themselves among aquatic organisms through a rich array of metabolites, including polysaccharides, polyphenols, pigments, peptides, amino acids, lipids, and sterols, though their composition varies by species^2,3^. Beyond these, algae supply vital vitamins and minerals, with extracts and biomass enhancing biological processes in plants and animals, particularly under stress^4^. However, the precise mechanisms by which algal bioactive compounds interact and exert synergistic effects on plant stress tolerance remain incompletely understood, representing a critical knowledge gap^5^.

Global algae production reached 35.82 million wet tonnes in 2019, with microalgae playing an increasingly important role^6^. Annual microalgal biomass output has expanded from about 10,000 tonnes a decade ago to nearly 60,000 tonnes today, driven by growing demand in the food, aquaculture, and nutraceutical industries, particularly in China, Japan, and the USA^7,8^. The global microalgae market, valued at USD 11.8 billion in 2023, is projected to surpass USD 25.4 billion by 2033, with an estimated annual growth rate of 8%^9^.

Despite significant interest in microalgae for biofuel production, high production costs have limited large-scale biodiesel adoption, resulting in most biomass being channeled toward higher-value applications^10^. Consequently, repurposing defatted biomass, a byproduct of biodiesel extraction, as a biostimulant presents an attractive avenue to enhance economic viability and promote circular bioeconomy principles. Despite growing interest, the potential of defatted microalgal biomass in improving cereal crop stress resilience remains underexplored, particularly in saline conditions.

Defatted microalgal biomass is rich in macronutrients, containing up to 10% nitrogen, 1–2% phosphorus, and 0.5–1% potassium^11,12^. Its application improves soil physical properties, stimulates beneficial microbial activity, and enhances nutrient availability and uptake, thereby reducing reliance on synthetic fertilizers^13,14^. Studies have demonstrated yield increases of 20–30% in leafy vegetables like lettuce following microalgal biofertilizer application^15–17^. Additionally, its high protein content (40–50%) supports its use as potentially sustainable livestock feed^11^. Compared to conventional fertilizers, microalgal biofertilizers often exhibit superior performance and contribute to lower greenhouse gas emissions, reducing emissions by up to 15% relative to chemical fertilizers^18,19^.

Among microalgae, *Chlorella* species are particularly valued for their high biomass productivity (0.08–0.42 g L¹ day¹ in BG-11 medium) and rich biochemical composition, surpassing other common genera such as *Scenedesmus*, *Diplosphaera*, and *Spirulina*^3,20^. *Chlorella* biomass typically contains 10–24% carbohydrates, 15–20% proteins, and 15–20% lipids, with defatted extracts showing promising biostimulant effects, particularly under drought and salinity stress^21,22^.

Barley (*Hordeum vulgare L*.) ranks as the fourth most widely cultivated cereal globally, with cultivation spanning approximately 48 million hectares across diverse climatic zones^23^. Its versatility supports critical industries including animal feed, brewing, and food production. However, barley yields have stagnated since 2000, lagging behind wheat and maize^23,24^. Salinity stress represents a significant constraint in many barley-growing regions, reducing yield by up to 50% and compromising grain quality. As a genetic model for cereal crop improvement, barley research offers insights into breeding stress-resilient varieties^25^. Soil salinity poses a major challenge to barley production, as well as to global agriculture more broadly, affecting nearly 50% of irrigated land. Salinity stress disrupts water uptake, nutrient absorption, and photosynthetic efficiency, often resulting in yield losses exceeding 70% under severe conditions^26–28^. As a salt-sensitive cereal crop, barley responds through physiological and biochemical adjustments, including osmolyte accumulation, enhanced antioxidant activity, and hormonal regulation^26,29^. However, despite advances in barley breeding, genetic engineering, and soil management, there is a continued need for potentially sustainable, cost-effective agronomic approaches to improve barley resilience in saline environments.

In this context, the present study explores the potential of defatted *Chlorella* sp. biomass as a potentially sustainable biostimulant to enhance barley growth and resilience under salinity stress. Although algal biostimulants have shown promising effects on plant development and stress tolerance, the mechanisms by which bioactive compounds from defatted microalgal biomass influence plant physiological and biochemical responses under saline conditions remain insufficiently understood. To our knowledge, few studies have investigated the use of defatted *Chlorella* biomass on barley, especially under saline conditions, making this approach both novel and potentially transformative for saline agriculture. Therefore, this study characterizes the bioactive compound profile of the defatted biomass and evaluates its effects on barley growth, photosynthetic performance, and nutrient accumulation under controlled saline conditions. The findings aim to advance potentially sustainable agricultural practices and improve the management of salinity-affected soils.

## Materials and methods

### Biomass preparation and defatting

The *Chlorella sp.* biomass used in this study was obtained as a monoculture from the Algae Research Unit, Agricultural Microbiology Department, Soils, Water and Environment Research Institute, Agricultural Research Center (ARC), Giza, Egypt. The culture was identified morphologically as *Chlorella sp.*, and although no accession number was available, its purity as a single algal species was confirmed before use. The harvested biomass was subsequently defatted and used for ethanol extraction. Lipids were extracted for biodiesel production using Soxhlet extraction with hexane as the solvent, following the method described by Sarat Chandra et al. (2015)^30^. For each extraction cycle, approximately 50 g of oven-dried biomass was loaded into a Soxhlet apparatus and refluxed with hexane for 8 h. at 68 °C. The defatted biomass was air-dried at 25 °C for 24 h and stored at 4 °C in airtight containers until further use.

### Ethanol extraction of bioactive compounds

Bioactive compounds were extracted from defatted *Chlorella* sp. biomass using 90% (v/v) ethanol at a solid-to-liquid ratio of 1:20 (w/v). The mixture was incubated at 60 °C for 24 h in a shaking incubator (150 rpm). After extraction, the mixture was filtered through Whatman No. 1 filter paper to remove residual cell debris, and the filtrate was concentrated using a rotary evaporator at 40 °C to approximately 25% of its original volume. The concentrated extract was then diluted with distilled water to obtain a working solution (1:100, v/v). This solution was used for seed soaking (12 h) and foliar application (5 mL per plant at 15-day intervals) according to the experimental treatments. Ethanol was selected as the extraction solvent due to its ability to efficiently extract a wide range of moderately polar bioactive compounds. In addition, its water miscibility and low toxicity make it suitable for preparing plant-applied formulations without phytotoxic effects.

### GC-MS analysis

The ethanol extract of defatted *Chlorella* sp. biomass was analyzed using a gas chromatography-mass spectrometry (GC-MS) system (Thermo Scientific, Austin, TX, USA) equipped with a TG-5MS capillary column (30 m × 0.25 mm i.d., 0.25 μm film thickness). The oven temperature was initially set at 50 °C and held for 2 min, then increased to 200 °C at a rate of 5 °C/min and held for an additional 2 min. Subsequently, the temperature was ramped to 290 °C at 30 °C/min and held for 2 min. The injector temperature was maintained at 270 °C, and the MS transfer line at 260 °C. Helium was used as the carrier gas at a constant flow rate of 1 mL/min. A 1 µL aliquot of the extract, diluted 1:10 in ethanol, was injected in split mode with a split ratio of 1:20 using an AS1300 autosampler, following a 3-minute solvent delay. Mass spectra were acquired under electron ionization (EI) at 70 eV in full scan mode, scanning a mass range of m/z 50–500. The ion source temperature was set to 200 °C. Compound identification was performed by comparing the obtained mass spectra and retention indices with those in the WILEY 09 and NIST 11 spectral libraries.

### Plant materials

Three barley (*Hordeum vulgare* L.) genotypes, Giza 123, Giza 132, and Giza 134, were obtained from the Barley Research Department, ARC. These genotypes were selected based on their contrasting responses to salinity stress and their agronomic relevance under Egyptian cultivation conditions. Seeds were surface-sterilized using a 2% sodium hypochlorite solution for 5 min, rinsed thoroughly three times with sterile distilled water, and then air-dried at room temperature before sowing.

### Hydroponic experiment under salinity stress

**Fig. 1:**
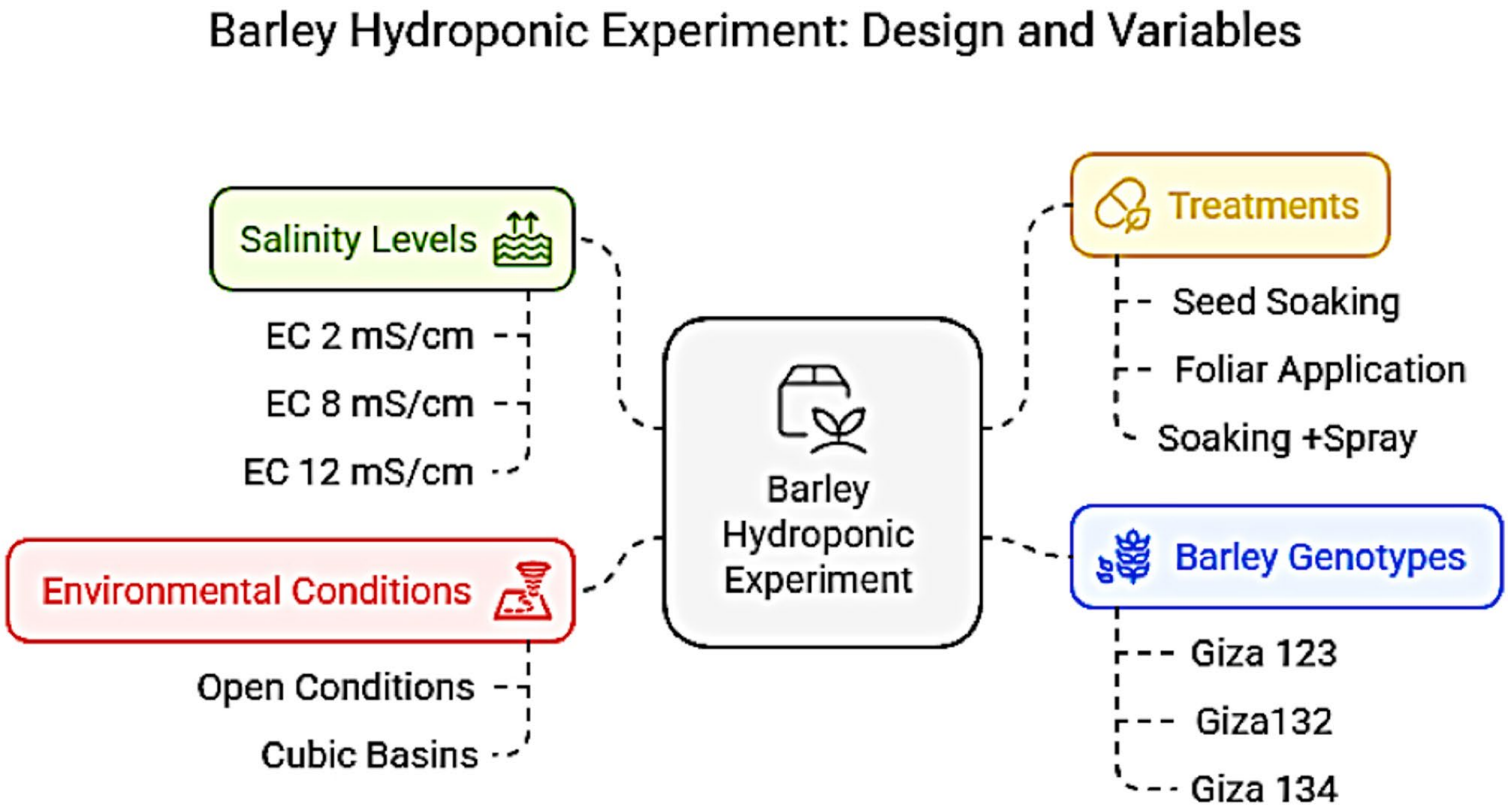
Schematic representation of the hydroponic experiment design.

Barley plants were grown hydroponically in floating beds with vermiculite-filled holes supported on a modified Hoagland nutrient solution^31^ to ensure optimal nutrient availability. The setup used cubic basins (70 × 70 × 50 cm) under open environmental conditions (average temperature 25 ± 3 °C, relative humidity 60–70%, natural daylight) to mimic field scenarios (Fig. 1). The nutrient solution composition followed Hoagland’s standard formulation (Supplementary Table 1), containing macronutrients (N, P, K, Ca, Mg, and S) and micronutrients (Fe, Mn, Zn, Cu, B, and Mo). Salinity stress was induced by supplementing the nutrient solution with NaCl and CaCl₂ (1:1 molar ratio) to achieve electrical conductivity (EC) levels of 2, 8, and 12 mS cm⁻¹, measured with an EC meter. Four treatments were applied: (1) seed soaking in ethanolic algal extract (1:100 dilution) for 12 h, (2) foliar spraying with the same extract every 15 days (5 mL/plant), (3) combined seed soaking and foliar spraying, and (4) a control with no extract, maintained under identical salinity and nutrient conditions. Barley seeds were first germinated for seven days in floating beds under non-saline conditions, after which the seedlings were transferred to nutrient solutions adjusted to the designated salinity levels (2, 8, and 12 mS cm⁻¹). The experiment followed a split-split plot design with three factors—genotypes (main plots), salinity levels (subplots), and treatments (sub-subplots)—replicated three times (*n* = 9 plants per treatment combination). Growth parameters were assessed after 60 days.

### Measurements and data collection

To assess the effects of defatted *Chlorella* sp. extract on barley (*Hordeum vulgare* L.) under salinity stress, physiological, biochemical, and agronomic parameters were quantified after 60 days of hydroponic growth.

**Agronomic traits** were quantified by measuring the dry weight of 100 grains (g) per plant, oven-dried at 70 °C for 48 h. until constant weight.

**Physiological measurements** included leaf temperature (°C) and chlorophyll fluorescence parameters, minimum fluorescence (F₀), maximum fluorescence (Fₘ), and the quantum yield of photosystem II (Fv/Fm, where Fv = Fₘ − F₀), were assessed using a FluorPen FP 100 (Photon Systems Instruments, Czech Republic) after 20 min of dark adaptation.

**Photosynthetic gas exchange parameters**, including differential transpiration rate (mmol H₂O/m²/s), net CO₂ assimilation rate (µmol CO₂/m²/s), and internal CO₂ concentration (µmol/mol), were measured using an LI-6400XT portable photosynthesis system (LI-COR Biosciences, Lincoln, NE, USA). Measurements were taken at midday (10:00–14:00) under natural light conditions.

**Biochemical analyses included**:

**Nutrient content** (N, P, K, and Na, all in %) in shoots and grains, determined using Kjeldahl digestion for nitrogen, colorimetric assay for phosphorus, and flame photometry for potassium and sodium.

**Grain protein content (%)** was calculated as *N* × 6.25, and **total protein** (mg/g dry weight) was quantified using the Bradford assay.

**Photosynthetic pigments**, including chlorophyll a, chlorophyll b, and carotenoids (mg/g fresh weight), were extracted with 80% acetone and measured spectrophotometrically at 663, 645, and 470 nm, respectively. The pigment concentrations were calculated using the following equations:

$${\text{Chlorophyll a }}\left( {{\mathrm{mg}}/{\text{g FW}}} \right){\text{ }}={\text{ }}({\mathrm{12}}.{\mathrm{7}} \times {{\mathrm{A}}_{{\mathrm{663}}}}){\text{ }} - {\text{ }}({\mathrm{2}}.{\mathrm{69}} \times {{\mathrm{A}}_{{\mathrm{645}}}})$$

$${\text{Chlorophyll b }}\left( {{\mathrm{mg}}/{\text{g FW}}} \right){\text{ }}={\text{ }}({\mathrm{22}}.{\mathrm{9}} \times {{\mathrm{A}}_{{\mathrm{645}}}}){\text{ }} - {\text{ }}({\mathrm{4}}.{\mathrm{68}} \times {{\mathrm{A}}_{{\mathrm{663}}}})$$

$${\text{Carotenoids }}\left( {{\mathrm{mg}}/{\text{g FW}}} \right){\text{ }}={\text{ }}[({\mathrm{1}}000 \times {{\mathrm{A}}_{470}}) - ({\mathrm{1}}.{\mathrm{82}} \times {\text{Chl a}}) - ({\mathrm{85}}.0{\mathrm{2}} \times {\text{Chl b}})]{\text{ }}/{\text{ 198}}$$

where *A₆₆₃*, *A₆₄₅*, and *A₄₇₀* represent absorbance at the respective wavelengths, and extinction coefficients were applied according to the standard method of Lichtenthaler and Wellburn^31^.

Measurements were conducted across all barley genotypes (Giza 123, Giza 132, Giza 134), salinity levels (2, 8, and 12 mS cm⁻¹), and treatments (seed soaking, foliar spray, combined application, and control). For each treatment combination, three plants were sampled per replicate (*n* = 9).

### Statistical analysis

The experiment followed a split-split plot design with three replicates per treatment combination (*n* = 9 plants total). The main plots were assigned to barley genotypes (Giza 123, Giza 132, Giza 134), subplots to salinity levels (2, 8, and 12 mS cm⁻¹), and sub-subplots to treatments (seed soaking, foliar spray, combined application, and control).

Data were subjected to a three-way analysis of variance (ANOVA) to evaluate the main effects and interactions of genotype, salinity level, and treatment on the measured physiological, biochemical, and agronomic parameters. The assumptions of normality and homogeneity of variance were tested using the Shapiro–Wilk and Levene’s tests, respectively.

When significant differences were detected, mean comparisons were conducted using the least significant difference (LSD) test at a significance level of *P* ≤ 0.05. All statistical analyses were performed using CoStat software (version 6.303; CoHort Software, Monterey, CA, USA).

## Results

### Bioactive compounds in defatted *Chlorella* sp. extract

**Fig. 2:**
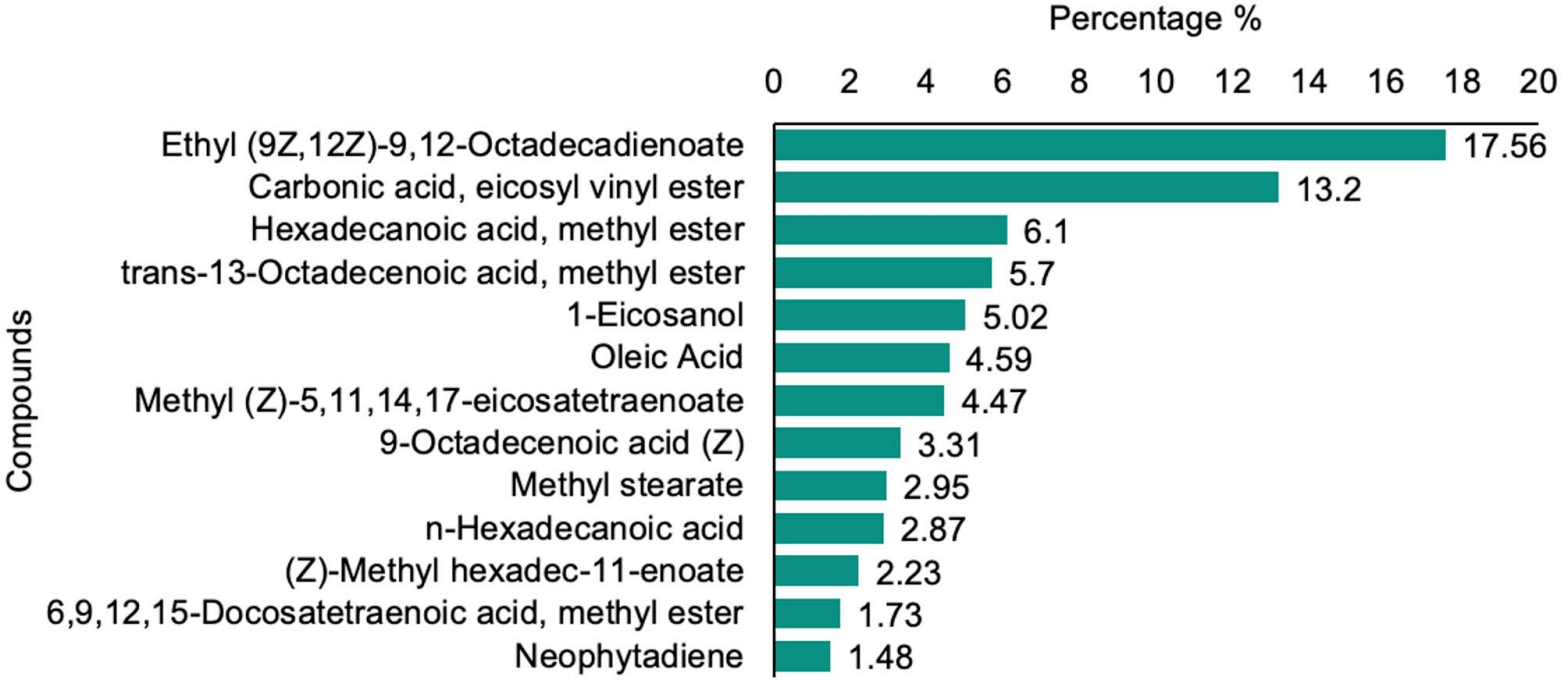
Relative abundance of bioactive compounds in defatted chlorella biomass as identified by GC-MS analysis.

Gas chromatography-mass spectrometry (GC-MS) analysis of the ethanol extract from defatted *Chlorella* sp. biomass identified 28 bioactive compounds (Table 1). The molecular weights of the detected compounds ranged from 128 to 536, with carbon chain lengths varying between 8 and 37 atoms. The most abundant compounds were ethyl (9Z,12Z)−9,12-octadecadienoate (18.72%), eicosyl vinyl carbonate (13.20%), hexadecanoic acid methyl ester (6.10%), and trans-13-octadecenoic acid methyl ester (5.70%) (Fig. 2). Other notable compounds included 1-eicosanol (10.92%), oleic acid (8.66%), and methyl (Z)−5,11,14,17-eicosatetraenoate (4.47%). Less abundant metabolites, such as methyl stearate (2.95%), n-hexadecanoic acid (2.87%), and 6,9,12,15-docosatetraenoic acid methyl ester (1.73%), were also detected. Molecular masses ranged from 128 to 536 Da, with carbon chain lengths spanning C8–C37. The identified compounds were predominantly fatty acids and their esters, known for their roles in modulating key plant physiological processes such as germination, root growth, and stress resilience.

**Table 1:** Bioactive compounds identified in the ethanol extract of defatted *Chlorella sp*. biomass by GC-MS.

No	Compound name	CAS	Formula	MW	RT	Area (%)
1	ETHYL (9Z,12Z)−9,12-OCTADECADIENOATE	544-35−4	C20H36O2	308	23.5	18.72
2	Carbonic acid, eicosyl vinyl ester		C23H44O3	368	15.6	13.2
3	Hexadecanoic acid, methyl ester	112-39−0	C17H34O2	270	19.6	6.1
4	trans-13-Octadecenoic acid, methyl ester		C19H36O2	296	22.4	5.7
5	1-Eicosanol	629-96−9	C20H42O	298	17.3	10.92
6	Oleic Acid	112-80−1	C18H34O2	282	23.1	8.66
7	Methyl(Z)−5,11,14,17-eicosatetraenoate	59149-01−8	C21H34O2	318	24.6	4.47
8	1-Hexadecanol	36653-82−4	C16H34O	242	13.6	6.56
9	9-OCTADECENOIC ACID (Z)	112-80−1	C18H34O2	282	25.3	4.07
10	Methyl stearate	112-61−8	C19H38O2	298	22.8	2.95
11	n-Hexadecanoic acid	1957/10/03	C16H32O2	256	20.3	2.87
12	(Z)-Methyl hexadec-11-enoate	822-05−9	C17H32O2	268	19.2	2.23
13	6,9,12,15-Docosatetraenoic acid, methyl ester	17364-34−0	C23H38O2	346	22.1	1.73
14	Neophytadiene	504-96−1	C20H38	278	18.1	2.45
15	3-(N, N-Dimethyllaurylammonio) propanesulfonate	14933-08−5	C17H37NO3S	335	12	1.37
16	2,6,10-Dodecatrien-1-ol,3,7,11-trimethyl-	4602-84−0	C15H26O	222	24.2	1.33
17	á-Sitosterol	83-46−5	C29H50O	414	30.2	2.18
18	1-DOCOSANOL	661-19−8	C22H46O	326	26.6	0.98
19	HI-OLEIC SAFFLOWER OIL	8001-23−8	C21H22O11	450	27.4	0.92
20	3,7,11,15-Tetramethyl-2-hexadecen-1-ol	102608-53−7	C20H40O	296	18.6	0.73
21	2-OCTANONE	111-13−7	C8H16O	128	15.8	0.58
22	17-Octadecynoic acid	34450-18−5	C18H32O2	280	20.8	1.11
23	ISOCHIAPIN B		C19H22O6	346	28.6	0.57
24	Caryophyllene	87-44−5	C15H24	204	10.3	0.55
25	[1,1’-Bicyclopropyl]−2-octanoic acid, 2’-hexyl-, methyl ester	56687-68−4	C21H38O2	322	18.2	0.37
26	Methyl tetradecanoate	124-10−7	C15H30O2	242	16.1	0.49
27	Hexadecanoic acid, 14-methyl-, methyl ester	2490-49−5	C18H36O2	284	21.2	0.4
28	1-CHLOROOCTADECANE	3386-33−2	C18H37Cl	288	13.8	0.38

### Impact of salinity, genotypes, and algal extract on growth parameters

**Fig. 3:**
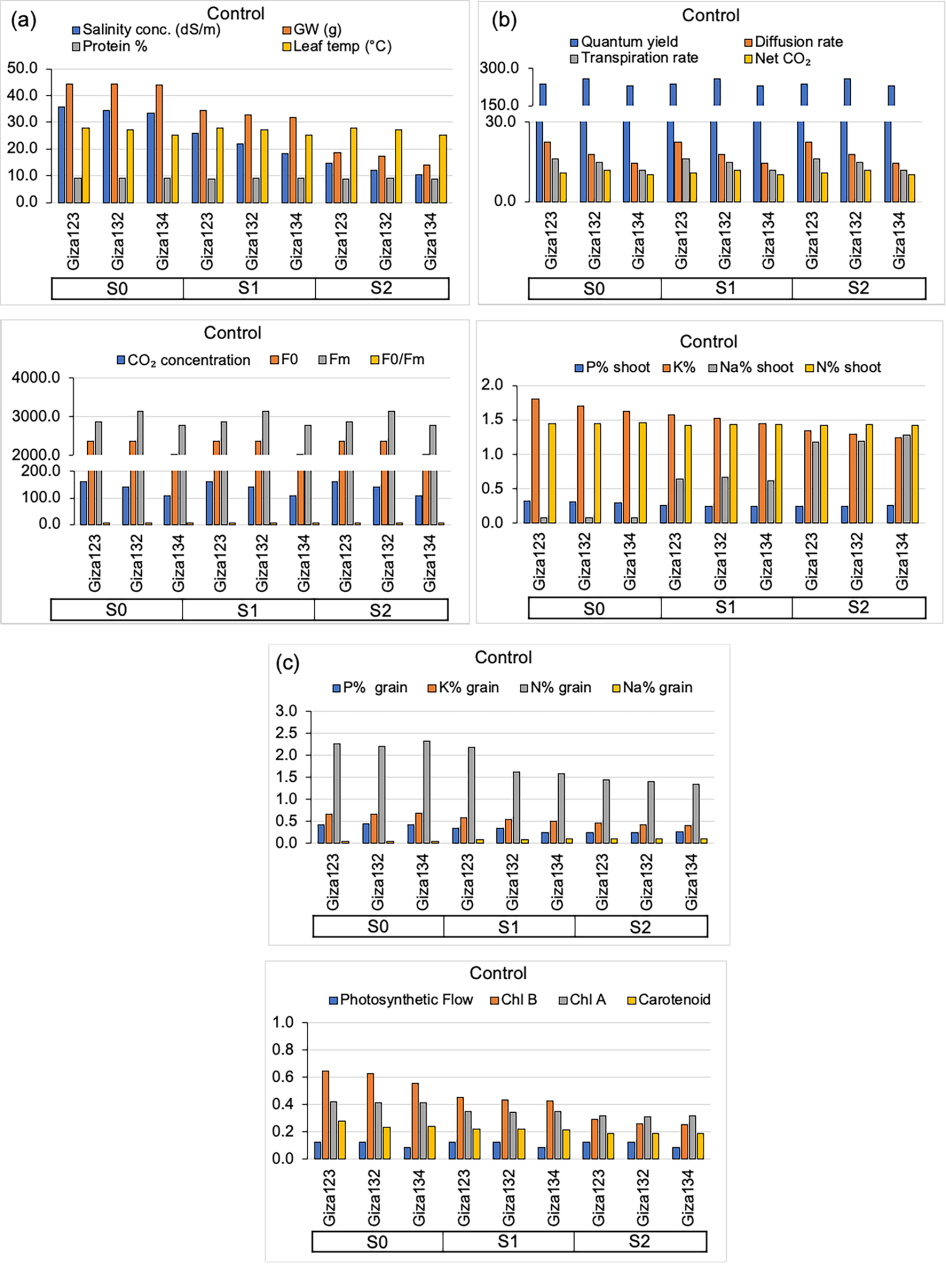
Mean phenotypic and physiological responses of barley genotypes to algal extract treatments (control). (**a**) Salinity concentration (mS cm⁻¹), grain weight (g), protein content (%), leaf temperature (°C), Quantum yield (ΦPSII, %), diffusion rate (mmol m⁻² s⁻¹), transpiration rate (mmol H₂O m⁻² s⁻¹), net CO₂ assimilation (µmol CO₂ m⁻² s⁻¹). (**b**) CO₂ concentration (µmol mol⁻¹), F₀, Fₘ, F₀/Fₘ, shoot P (%), K (%), Na (%), and N (% (**c**) Grain P (%), K (%), Na (%), and N (%), Photosynthetic Flow, chlorophyll b (Chl b), chlorophyll a (Chl a), and carotenoid content.

Salinity stress had a detrimental effect on grain weight and electron transport rate (ETR) in barley, while leaf temperature remained unchanged (Table 2). Grain weight decreased from 35.82 g at 0 mS cm⁻¹ (S0) to 23.16 g at moderate salinity (S1) and further declined to 13.14 g under high salinity (S2) (Fig. 3a). Electron transport rate (ETR) followed a similar trend, decreasing from 45.17 in S0 to 34.88 in S1 and 18.39 in S2. The differences in grain weight and electron transport rate (ETR) among salinity treatments were statistically significant (*p* < 0.05).

**Table 2:** Effects of salinity, genotypes, and algal extracts on grain weight, photosynthesis, and leaf temperature.

Treatments	Salinity concentration (dS/m)	Grains weight (g)	Photosynthetic Flow	Leaf temp (°C)
**(A) Main (Salinity)**				
S0	35.82	45.17	0.12	26.65
S1	23.16	34.88	0.12	26.65
S2	13.14	18.39	0.12	26.65
L.S.D 0.05	0.41**	0.53**	n.s	n.s
**(B) Sub main (genotypes)**				
Giza 123	26.09	34.82	0.13	27.93
Giza 132	23.91	32.59	0.12	26.51
Giza 134	22.13	31.02	0.1	25.51
L.S.D. 0.05	0.17**	0.29**	0.004**	0.05**
**(C) Sub sub main (algal extracts)**				
Control	22.94	31.28	0.11	26.77
Spray	23.94	32.74	0.12	26.75
Soaking	24.3	33.09	0.12	27.03
Soaking +spray	24.99	34.13	0.11	26.03
L.S.D. 0.05	0.28**	0.25**	0.003**	0.21**
(A*B) L.S.D. 0.05	0.38**	0.38**	n.s	n.s
(A*C) L.S.D. 0.05	n.s	0.43**	n.s	n.s
(B*C) L.S.D. 0.05	0.48*	0.44**	0.003**	0.36**
(A*B*C) L.S.D. 0.05	0.45**	0.76**	0.01**	0.63**
S0 = 2dsm^− 1^ S0 = 6dsm^− 1^ S0 = 12dsm^− 1^				

Among the barley genotypes, Giza 123 exhibited the highest grain weight (26.09 g), followed by Giza 132 (23.91 g) and Giza 134 (22.13 g). Similarly, Giza 123 showed the highest electron transport rate (ETR) (34.82), while Giza 132 and Giza 134 had lower values (32.59 and 31.02, respectively). Giza 123 also exhibited the highest leaf temperature (27.93 °C), whereas Giza 134 recorded the lowest (25.51 °C). These genotype-based differences were statistically significant (*p* < 0.05), indicating variation in salinity tolerance among the tested cultivars.

The combined soaking and foliar spray treatment resulted in the highest grain weight (24.99 g) and electron transport rate (ETR) (34.13), whereas the control treatment recorded the lowest values (22.94 g and 31.28, respectively) (Fig. 6). Single applications (either spraying or soaking) led to moderate improvements in these parameters. Leaf temperature was slightly influenced by algal treatment, with the soaking treatment showing a marginal increase (27.03 °C) compared to the control (26.77 °C).

The interaction effects revealed significant genotype × algal application (B × C) and salinity × genotype (A × B) interactions for grain weight and electron transport rate (ETR), suggesting that different barley cultivars responded variably to both salinity stress and algal treatments. However, salinity × algal application (A × C) interaction effects were not significant for grain weight. A significant interaction was observed for electron transport rate (ETR), indicating that the response to algal application varied under different salinity levels.

These results demonstrate that salinity stress negatively affects barley growth and photosynthetic efficiency, but the application of defatted *Chlorella* biomass as a biostimulant can mitigate some of these effects, particularly through combined soaking and foliar spraying. Giza 123 was the most resilient genotype, exhibiting the highest grain yield and photosynthetic efficiency under salinity stress.

### Effects of salinity, genotypes, and algal extract on quantum yield, diffusion rate, transpiration rate, and Net CO₂ assimilation

Salinity treatments (2, 8, and 12 mS cm⁻¹) did not significantly influence quantum yield, diffusion rate, transpiration rate, or net CO₂ assimilation in barley (Fig. 3b; Table 3). The values for these parameters remained relatively stable across salinity levels, indicating that the imposed salt stress had no substantial effect on these specific physiological traits.

**Table 3:** Effects of salinity, genotypes, and algal extracts on quantum yield, diffusion rate, transpiration rate, and Net CO₂ assimilation in Barley.

Treatments	Quantum yield (ΦPSII) %	Diffusion rate (mmol m⁻² s⁻¹)	Transpiration rate (mmol H₂O m⁻² s⁻¹)	Net CO₂ assimilation (µmol CO₂ m⁻² s⁻¹)
**(A) Main (Salinity)**				
S0	256.76	18.63	14.01	11.55
S1	256.76	18.63	14.01	11.55
S2	256.76	18.63	14.01	11.55
L.S.D 0.05	n.s	n.s	n.s	n.s
**(B) Sub main (genotypes)**				
Giza 123	247.23	19.56	14.77	11.13
Giza 132	270.63	17.21	14.35	12.18
Giza 134	252.41	19.12	12.91	11.36
L.S.D. 0.05	8.91**	0.79**	0.63**	0.4**
**(C) Sub sub main (algal extracts)**				
Control	241.67	18.24	14.19	10.88
Spray	254.2	19.97	14.35	11.44
Soaking	268.52	17.54	13.91	12.08
Soaking +spray	262.64	18.77	13.58	11.82
L.S.D. 0.05	11.15**	1.48**	n.s	0.5**
(A*B) L.S.D. 0.05	n.s	n.s	n.s	n.s
(A*C) L.S.D. 0.05	n.s	n.s	n.s	n.s
(B*C) L.S.D. 0.05	12.3**	2.57**	2.22**	0.87**
(A*B*C) L.S.D. 0.05	33.45**	4.45**	3.85**	1.51**
S0 = 2dsm^− 1^ S0 = 6dsm^− 1^ S0 = 12dsm^− 1^				

In contrast, significant variations were detected among the barley genotypes for all measured photosynthetic parameters (Table 3). Giza 132 exhibited the highest chlorophyll fluorescence (quantum yield = 270.63 × 10⁻³, arbitrary units) and the greatest net CO₂ assimilation (12.18 µmol CO₂ m⁻² s⁻¹), while Giza 123 recorded the lowest values (247.23 × 10⁻³ and 11.13 µmol CO₂ m⁻² s⁻¹, respectively). Regarding gas exchange traits, Giza 123 showed the highest diffusion rate (19.56 mmol m⁻² s⁻¹) and transpiration rate (14.77 mmol H₂O m⁻² s⁻¹), whereas Giza 134 exhibited the lowest rates (19.12 mmol m⁻² s⁻¹ and 12.91 mmol H₂O m⁻² s⁻¹, respectively). These differences highlight the genetic variability in photosynthetic performance among the tested cultivars.

Application of the defatted Chlorella extract significantly influenced quantum yield, diffusion rate, and net CO₂ assimilation, but had no significant effect on transpiration rate (Table 3). Among the treatments, seed soaking produced the highest quantum yield (268.52 × 10⁻³) and net CO₂ assimilation (12.08 µmol CO₂ m⁻² s⁻¹), followed by foliar spray (quantum yield = 254.20 × 10⁻³; net CO₂ assimilation = 11.44 µmol CO₂ m⁻² s⁻¹). The control consistently produced the lowest values for quantum yield (241.67 × 10⁻³) and net CO₂ assimilation (10.88 µmol CO₂ m⁻² s⁻¹) (Fig. 3), confirming the stimulatory effect of the algal extract on photosynthetic performance.

Significant interaction effects were observed between genotype and algal treatment for all photosynthetic parameters. Additionally, the three-way interaction among salinity, genotype, and algal application was significant, particularly for quantum yield (F = 33.45), diffusion rate (F = 4.45), transpiration rate (F = 3.85), and net CO₂ assimilation (F = 1.51). These results demonstrate that the combined effects of genotype, salinity, and algal biostimulant application collectively modulate the photosynthetic responses of barley under saline conditions.

### Impact of salinity, genotypes, and algal extracts on chlorophyll fluorescence parameters

Salinity stress had no significant effect on initial CO₂ concentration, minimum fluorescence (F₀), maximum fluorescence (FM), or the FV/FM ratio in barley (Fig. 3; Table 4). Across all salinity levels (S0, S1, and S2), these parameters remained unchanged, indicating that salinity did not significantly alter chlorophyll fluorescence characteristics or initial CO₂ availability.

**Table 4:** Effects of Salinity, genotypes, and algal extracts on Initial CO₂ concentration, minimum fluorescence (F₀), maximum fluorescence (Fm), and F₀/Fm ratio in barley.

Treatments	CO₂ concentration (µmol mol⁻¹)	F_0_	Fm	F0/Fm
**(A) Main (Salinity)**				
S0	141	2343.56	3107.78	0.76
S1	141	2343.56	3107.78	0.76
S2	141	2343.56	3107.78	0.76
L.S.D 0.05	n.s	n.s	n.s	n.s
**(B) sub main (genotypes)**				
Giza 123	149.99	2353.58	2994.83	0.8
Giza 132	136.63	2388.08	3274.08	0.73
Giza 134	136.37	2289	3054.42	0.75
L.S.D. 0.05	3.55**	26.41**	106.**	0.002**
**(C) sub sub main (algal extracts)**				
Control	136.99	2244.33	2926.89	0.77
Spray	146.95	2368.89	3077.33	0.78
Soaking	140.34	2397.78	3249.22	0.74
Soaking +spray	139.71	2363.22	3177.67	0.75
L.S.D. 0.05	3.57**	65.76**	133.8**	0.017**
(A*B) L.S.D. 0.05	n.s	n.s	n.s	n.s
(A*C) L.S.D. 0.05	n.s	n.s	n.s	n.s
(B*C) L.S.D. 0.05	14.23**	128.5**	198.76**	0.03**
(A*B*C) L.S.D. 0.05	24.6**	157.27**	241.4**	0.05**
S0 = 2dsm^− 1^ S0 = 6dsm^− 1^ S0 = 12dsm^− 1^				

Significant differences were observed among the barley genotypes in all measured parameters. Giza 123 recorded the highest initial CO₂ concentration (149.99 ppm) and FV/FM ratio (0.80), whereas Giza 132 showed the lowest values (136.63 ppm and 0.73, respectively). However, Giza 132 exhibited the highest FM (3274.08), suggesting enhanced photosystem II (PSII) maximum fluorescence efficiency, while Giza 123 had the lowest value (2994.83). These statistically significant differences (*p* < 0.05) highlight the genetic variability among the tested cultivars in terms of fluorescence response and photosynthetic capacity.

**Fig. 4:**
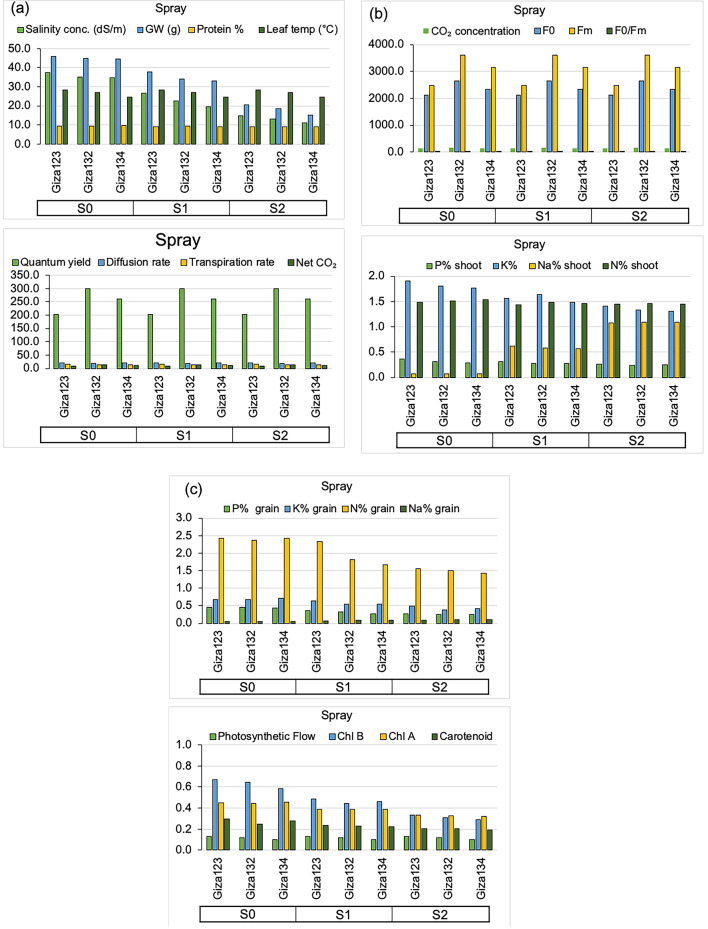
Mean phenotypic and physiological responses of barley genotypes to algal extract treatments (foliar spray) under varying salinity conditions. (**a**) Salinity concentration (mS cm⁻¹), grain weight (g), protein content (%), leaf temperature (°C), Quantum yield (ΦPSII, %), diffusion rate (mmol m⁻² s⁻¹), transpiration rate (mmol H₂O m⁻² s⁻¹), net CO₂ assimilation (µmol CO₂ m⁻² s⁻¹). (**b**) CO₂ concentration (µmol mol⁻¹), F₀, Fₘ, F₀/Fₘ, shoot P (%), K (%), Na (%), and N (%). (**c**) Grain P (%), K (%), Na (%), and N (%), Photosynthetic Flow, chlorophyll b (Chl b), chlorophyll a (Chl a), and carotenoid content.

The foliar spray treatment led to the highest initial CO₂ concentration (146.95 ppm) (Fig. 4), while the control treatment had the lowest (136.99 ppm). Minimum fluorescence (F₀) was highest with the soaking treatment (2397.78), indicating improved energy capture by PSII, whereas the control recorded the lowest F₀ (2244.33). Similarly, FM reached its maximum value under the soaking treatment (3249.22), suggesting improved fluorescence efficiency. The FV/FM ratio varied among treatments, with the highest value observed in the spray treatment (0.78) and the lowest in the soaking treatment (0.74), reflecting differences in photochemical efficiency.

Interaction analyses revealed significant genotype × algal application (B × C) interactions for all measured parameters, indicating that the cultivars responded differently to algal treatments. In contrast, the salinity × genotype (A × B) and salinity × algal application (A × C) interactions were not significant, suggesting that salinity did not significantly influence the genotype or algal treatment effects. However, a significant three-way interaction (A × B × C) was observed for all parameters, pointing to a complex interplay between salinity, genotype, and algal application in modulating chlorophyll fluorescence responses.

Overall, these findings suggest that salinity stress had no direct impact on chlorophyll fluorescence or initial CO₂ concentration. However, the application of defatted *Chlorella* biomass, particularly through seed soaking, enhanced PSII efficiency and CO₂ availability. Giza 132 demonstrated superior fluorescence performance, making it a promising candidate for further investigations into photosynthetic resilience under abiotic stress conditions.

### Impact of genotypes and algal extracts on macronutrient accumulation in barley shoots

Salinity had a significant impact on the macronutrient content in barley shoots (Table 5). As salinity increased from S0 (control) to S2 (high salinity), P, K, Na, and N contents decreased. Specifically, P content decreased from 0.34 mg g⁻¹ at S0 to 0.26 mg g⁻¹ at S2, while K content declined from 1.85 mg g⁻¹ to 1.36 mg g⁻¹ across the same salinity gradient. In contrast, Na content increased sharply under high salinity, rising from 0.07 mg g⁻¹ at S0 to 1.10 mg g⁻¹ at S2. These trends indicate that elevated salinity exacerbates ionic imbalance in barley shoots, promoting Na accumulation while reducing the availability of essential nutrients such as P and K.

**Table 5:** Macronutrient content (P, K, Na, and N) in barley shoots as affected by salinity, genotypes, and algal extracts.

Treatments	*P* mg g^− 1^ shoot	K mg g^− 1^ shoot	Na mg g^− 1^ shoot	*N*% shoot
**(A) Main (Salinity)**				
S0	0.34	1.85	0.07	1.52
S1	0.29	1.56	0.58	1.47
S2	0.26	1.36	1.1	1.47
L.S.D 0.05	0.009**	0.086**	0.03**	0.018**
**(B) Sub main (genotypes)**				
Giza 123	0.32	1.66	0.59	1.48
Giza 132	0.29	1.61	0.59	1.5
Giza 134	0.29	1.5	0.58	1.49
L.S.D. 0.05	0.005**	0.07**	n.s	0.014**
**(C) Sub sub main (algal extracts)**				
Control	0.27	1.51	0.65	1.43
Spray	0.29	1.58	0.58	1.47
Soaking	0.3	1.62	0.58	1.51
Soaking +spray	0.32	1.65	0.54	1.54
L.S.D. 0.05	0.009**	0.08**	0.02*	0.012**
(A*B) L.S.D. 0.05	0.014**	0.11**	0.03**	0.019**
(A*C) L.S.D. 0.05	0.016**	0.14**	0.03**	0.022**
(B*C) L.S.D. 0.05	n.s	0.14**	n.s	0.02*
(A*B*C) L.S.D. 0.05	0.03**	0.24**	0.07**	0.037**
S0 = 2dsm^− 1^ S0 = 6dsm^− 1^ S0 = 12dsm^− 1^				

Significant genotypic differences were observed in macronutrient accumulation. Giza 123 recorded the highest P (0.32 mg g⁻¹) and K (1.66 mg g⁻¹) contents, while Giza 134 had the lowest levels of P (0.29 mg g⁻¹) and K (1.50 mg g⁻¹). Nitrogen content did not vary significantly among the cultivars, although Giza 132 exhibited the highest N content (1.50%), compared to 1.48% in Giza 123. These findings suggest that genotypic variation plays a role in nutrient uptake efficiency, with Giza 132 showing a slight advantage in nitrogen assimilation.

**Fig. 5:**
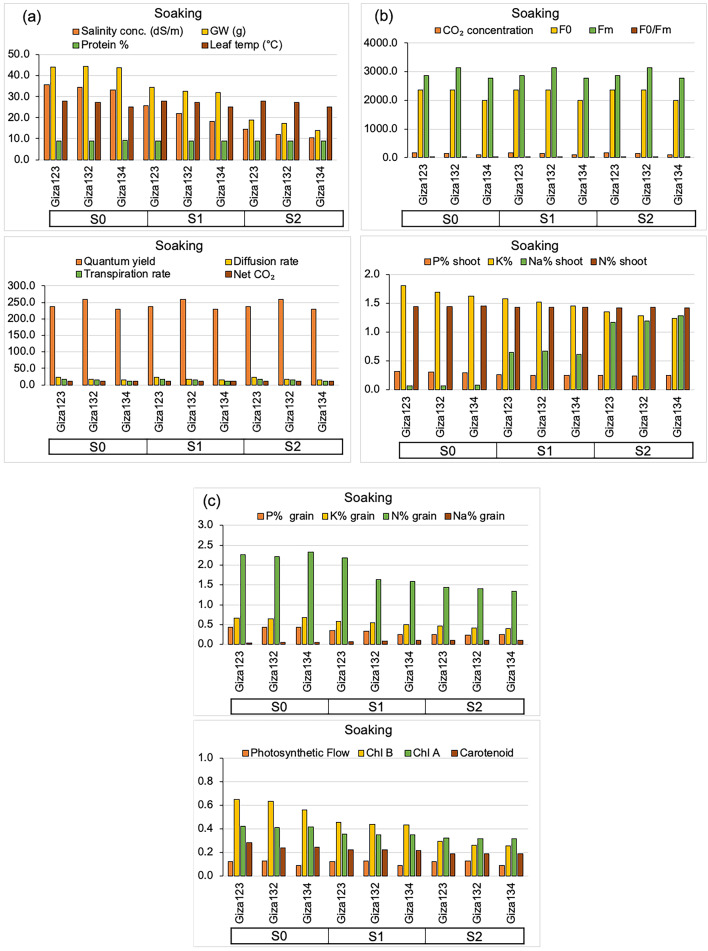
Mean phenotypic and physiological responses of barley genotypes to algal extract treatments (seed soaking) under varying salinity conditions. (**a**) Salinity concentration (mS cm⁻¹), grain weight (g), protein content (%), leaf temperature (°C), Quantum yield (ΦPSII, %), diffusion rate (mmol m⁻² s⁻¹), transpiration rate (mmol H₂O m⁻² s⁻¹), net CO₂ assimilation (µmol CO₂ m⁻² s⁻¹). (**b**) CO₂ concentration (µmol mol⁻¹), F₀, Fₘ, F₀/Fₘ, shoot P (%), K (%), Na (%), and N (%). (**c**) Grain P (%), K (%), Na (%), and N (%), Photosynthetic Flow, chlorophyll b (Chl b), chlorophyll a (Chl a), and carotenoid content.

**Fig. 6:**
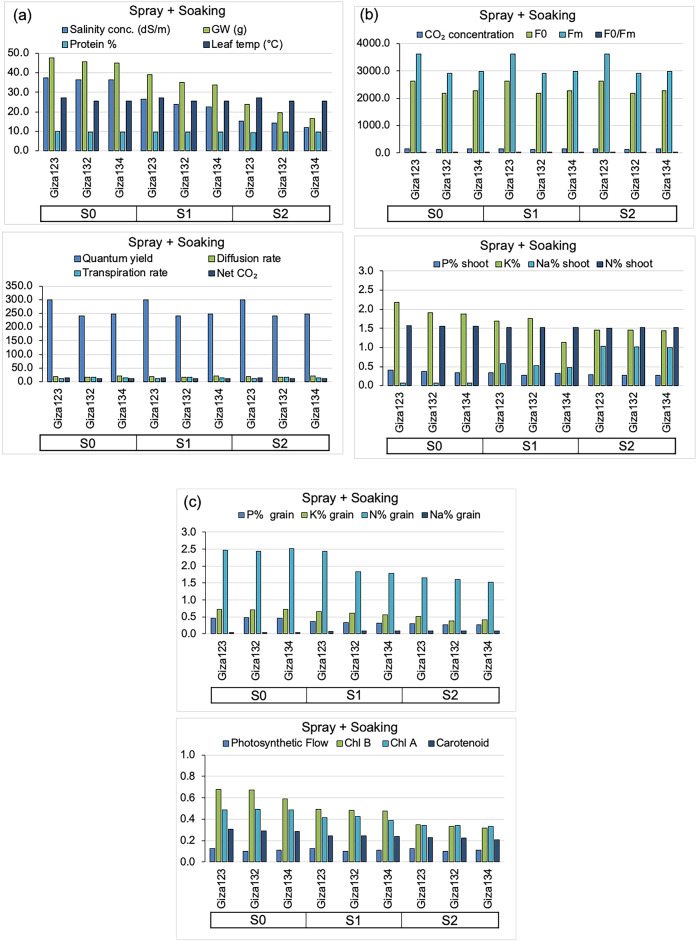
Mean phenotypic and physiological responses of barley genotypes to algal extract treatments (combined seed soaking and foliar spray) under varying salinity conditions. (**a**) Salinity concentration (mS cm⁻¹), grain weight (g), protein content (%), leaf temperature (°C), Quantum yield (ΦPSII, %), diffusion rate (mmol m⁻² s⁻¹), transpiration rate (mmol H₂O m⁻² s⁻¹), net CO₂ assimilation (µmol CO₂ m⁻² s⁻¹). (**b**) CO₂ concentration (µmol mol⁻¹), F₀, Fₘ, F₀/Fₘ, shoot P (%), K (%), Na (%), and N (%). (**c**) Grain P (%), K (%), Na (%), and N (%), Photosynthetic Flow, chlorophyll b (Chl b), chlorophyll a (Chl a), and carotenoid content.

Algal extract application had a significant positive effect on macronutrient accumulation. The soaking treatment was particularly effective, resulting in the highest P (0.32 mg g⁻¹), K (1.65 mg g⁻¹), and N (1.54%) contents, indicating enhanced nutrient uptake (Fig. 5). The spray treatment also improved nutrient accumulation, especially K (1.58 mg g⁻¹) and N (1.47%) contents, although to a lesser extent than soaking. The control treatment, which received no algal extract, consistently showed the lowest nutrient levels. Notably, Na content was lowest in the combined soaking + spray treatment (0.54 mg g⁻¹) (Fig. 6), suggesting that algal application may help mitigate salinity-induced Na accumulation.

Significant three-way interaction effects (A × B × C) were observed among salinity, genotype, and algal treatment for all measured macronutrients, including P, K, Na, and N. These interactions underscore the complex interplay between environmental stress, genetic background, and biostimulant application in determining nutrient dynamics in barley shoots.

Overall, the results demonstrate that algal treatments, particularly seed soaking with *Chlorella* extract can effectively enhance macronutrient uptake and alleviate some of the negative impacts of salinity stress. Furthermore, the observed genotypic variation highlights the potential for selecting and cultivating barley cultivars with superior nutrient assimilation capacity and greater tolerance to saline environments. Among the tested genotypes, Giza 132 exhibited a relatively higher efficiency in nitrogen uptake, making it a promising candidate for saline agriculture.

### Impact of genotypes and algal extracts on grain macronutrient composition

The macronutrient composition of barley grains (N, P, K, and Na) was significantly influenced by salinity level, genotype, and algal extract application (Table 6). Increasing salinity markedly reduced the concentrations of essential nutrients in the grains. Phosphorus content decreased by about 42% (from 0.45% at S0 to 0.26% at S2), potassium by 39% (from 0.70% to 0.43%), and nitrogen by 37% (from 2.38% to 1.50%). In contrast, sodium content increased by 150% (from 0.04% at S0 to 0.10% at S2), indicating ion imbalance under high salinity stress. These findings indicate that salinity stress reduces the accumulation of vital nutrients in barley grains and promotes sodium accumulation, which is generally harmful to plant growth and grain quality.

**Table 6:** Effects of salinity, genotypes, and algal extracts on grain macronutrient composition (P, K, N, and Na).

Treatments	*N*%	*P*%	K%	Na%
**(A) Main (Salinity)**				
S0	2.38	0.45	0.7	0.04
S1	1.92	0.32	0.58	0.08
S2	1.5	0.26	0.43	0.1
L.S.D 0.05	0.031**	0.0068**	0.006**	0.002**
**(B) Sub main (genotypes)**				
Giza 123	2.08	0.36	0.6	0.07
Giza 132	1.87	0.34	0.55	0.07
Giza 134	1.85	0.33	0.55	0.08
L.S.D. 0.05	0.024**	0.0065**	0.007**	0.0021**
**(C) Sub sub main (algal extracts)**				
Control	1.82	0.33	0.54	0.08
Spray	1.95	0.34	0.57	0.07
Soaking	1.94	0.34	0.57	0.07
Soaking +spray	2.03	0.36	0.59	0.07
L.S.D. 0.05	0.027**	0.0086**	0.008**	0.0016**
(A*B) L.S.D. 0.05	0.039**	0.013**	0.012**	0.0023**
(A*C) L.S.D. 0.05	n.s	n.s	0.014**	n.s
(B*C) L.S.D. 0.05	n.s	0.015**	0.014**	0.0028**
(A*B*C) L.S.D. 0.05	0.08**	0.026**	0.024	0.0047**
S0 = 2dsm^− 1^ S0 = 6dsm^− 1^ S0 = 12dsm^− 1^				

Among the tested barley genotypes, Giza 123 consistently showed the highest P (0.36%) and N (2.08%) contents, followed by Giza 132 and Giza 134, which exhibited slightly lower but comparable nutrient levels. Similarly, K and Na contents were highest in Giza 123, while Giza 132 and Giza 134 had comparable potassium (0.55%) and sodium (0.07%) levels. This suggests that Giza 123 may possess a superior capacity for nutrient accumulation in grains relative to the other genotypes.

The combined soaking + spraying treatment produced the highest P (0.36%) and K (0.59%) contents, as well as increased N content (2.03%), compared to other treatments. The control treatment consistently recorded the lowest P (0.33%) and K (0.54%) contents (Fig. 6). Na content remained relatively stable across all algal treatments, ranging from 0.07% to 0.08%. These results highlight that the synergistic application of algal extracts through soaking and foliar spraying can effectively promote the accumulation of key macronutrients in barley grains.

Significant three-way interactions (salinity × genotype × algal treatment, A × B × C) were observed for P, K, and N contents, indicating that the influence of algal treatments on nutrient accumulation is dependent on both salinity level and barley genotype. Na content exhibited significant interaction effects only between genotype and algal treatment (B × C).

In summary, algal extract application, particularly the combined soaking and spraying method, improves the macronutrient composition of barley grains. Among genotypes, Giza 123 showed the greatest capacity for nutrient accumulation under various conditions. However, salinity stress adversely affected grain nutrient content, underscoring the potential of algal treatments to mitigate the negative effects of salinity on grain quality.

### Influence of genotypes and algal extracts on grain protein content and photosynthetic pigments

Protein content and photosynthetic pigments (Chlorophyll A, Chlorophyll B, and Carotenoids) in barley grains were significantly influenced by salinity, genotype, and algal extract application (Table 7). With increasing salinity, all measured traits showed a significant decline. Protein content decreased from 9.49% at S0 to 9.19% at S2 (Fig. 3c). Similarly, Chlorophyll B content dropped from 0.63 mg/g to 0.31 mg/g, Chlorophyll A from 0.45 mg/g to 0.33 mg/g, and carotenoid content decreased from 0.28 mg/g at S0 to 0.20 mg/g at S2. These findings indicate that salinity stress negatively affects both protein synthesis and the accumulation of photosynthetic pigments in barley grains.

**Table 7:** Effects of salinity, genotypes, and algal extracts on grain protein content and photosynthetic pigments (Chlorophyll A, Chlorophyll B, and Carotenoids).

Treatments	Protein %	Chl B mg/g/FW	Chl A mg/g/FW	Carotenoid mg/g/FW
**(A) Main (Salinity)**				
S0	9.49	0.63	0.45	0.28
S1	9.22	0.46	0.38	0.23
S2	9.19	0.31	0.33	0.2
L.S.D 0.05	0.11**	0.013**	0.005**	0.005**
**(B) Sub main (genotypes)**				
Giza 123	9.22	0.49	0.39	0.25
Giza 132	9.35	0.47	0.39	0.23
Giza 134	9.33	0.44	0.38	0.23
L.S.D. 0.05	0.089**	0.0034**	0.0025**	0.005**
**(C) Sub sub main (algal extracts)**				
Control	8.96	0.44	0.36	0.22
Spray	9.21	0.47	0.39	0.24
Soaking	9.44	0.47	0.39	0.24
Soaking +spray	9.6	0.49	0.41	0.25
L.S.D. 0.05	0.078**	0.0069**	0.005**	0.006**
(A*B) L.S.D. 0.05	n.s	0.01**	0.008**	0.0088**
(A*C) L.S.D. 0.05	n.s	n.s	0.009**	n.s
(B*C) L.S.D. 0.05	0.13**	n.s	n.s	0.01**
(A*B*C) L.S.D. 0.05	0.23**	0.021*	0.15**	0.017**
S0 = 2dsm^− 1^ S0 = 6dsm^− 1^ S0 = 12dsm^− 1^				

Among the genotypes, Giza 132 exhibited the highest protein content (9.35%), followed closely by Giza 134 (9.33%) and Giza 123 (9.22%). In terms of pigment content, Giza 123 showed the highest levels of Chlorophyll B (0.49 mg/g) and Chlorophyll A (0.39 mg/g), while Giza 134 recorded the lowest values for both chlorophylls and carotenoids. This suggests that Giza 132 may have superior protein accumulation under various salinity conditions, whereas Giza 123 excels in photosynthetic pigment accumulation.

The soaking + spray treatment resulted in the highest protein content (9.60%), followed by soaking (9.44%) and spraying (9.21%), with the control treatment showing the lowest protein content (8.96%). Similarly, the soaking + spraying treatment resulted in the highest Chlorophyll B (0.49 mg/g), Chlorophyll A (0.41 mg/g), and carotenoid content (0.25 mg/g), while the control treatment had the lowest pigment levels (Chlorophyll B = 0.44 mg/g, Chlorophyll A = 0.36 mg/g, carotenoids = 0.22 mg/g) (Fig. 6). These results underscore the beneficial effects of algal extracts, particularly when applied through both soaking and spraying, on improving protein and pigment levels in barley grains.

Significant interaction effects were observed between salinity, genotype, and algal application (A × B × C) for protein content and photosynthetic pigments. For chlorophylls and carotenoids, significant interactions occurred between genotype and algal treatment (B × C), whereas protein content showed significant interactions across all three factors (A × B × C). These interactions indicate that genotype and algal treatment modulate the impact of salinity on barley grain protein and pigment accumulation.

In conclusion, algal extract application, especially the combined soaking and spraying method, enhances both protein content and photosynthetic pigments in barley grains. Giza 132 showed superior protein accumulation, while Giza 123 performed best in chlorophyll and carotenoid levels. Although salinity stress reduced grain quality, algal treatments effectively mitigated these negative effects, offering a promising approach to improve barley productivity under salinity stress.

Overall, the combined seed soaking and foliar spray treatment consistently produced the highest improvements across physiological and growth parameters, confirming its superior efficacy in mitigating salinity-induced stress compared with individual treatments.

## Discussion

Recent studies have demonstrated that *Chlorella spp*. and microalgal extracts enhance plant tolerance to salinity through multiple physiological and biochemical mechanisms. These include stimulation of antioxidant defense systems (e.g., SOD, CAT, APX), reduction of oxidative damage, modulation of hormone signaling pathways, and increased accumulation of osmoprotectants such as proline and glycine betaine, leading to improved ion homeostasis and K⁺/Na⁺ balance under salt stress^4,32,33^. In addition, algal treatments have been associated with enhanced photosynthetic performance, nutrient uptake, and biomass production in several crops under saline conditions, supporting their role as effective biostimulants. These findings are consistent with the results of the present study, confirming the potential of Chlorella-based extracts in improving plant growth and stress resilience.

The present study aimed to assess the effects of salinity stress on barley growth and photosynthetic performance, as well as the potential mitigating effect of algal extract from defatted *Chlorella* sp. on these parameters. Our findings provide compelling evidence that algal extracts, particularly when applied via both soaking and foliar spray, can ameliorate the adverse effects of salinity stress on growth and photosynthetic performance in barley.

### Composition of bioactive compounds and their potential physiological roles

The GC–MS analysis revealed that the ethanol extract from defatted *Chlorella* sp. biomass contained 28 bioactive compounds, primarily fatty acids, esters, and alcohols. Among these, ethyl (9Z,12Z)−9,12-octadecadienoate, eicosyl vinyl carbonate, and hexadecanoic acid methyl ester were dominant. These metabolites are known to play key roles in plant growth regulation and abiotic stress tolerance. Fatty acids and their derivatives act as precursors of signaling molecules such as jasmonates and contribute to maintaining membrane fluidity and integrity under saline conditions^33,34^. Additionally, long-chain esters and alcohols enhance plant stress resilience by reducing ion leakage, protecting photosynthetic pigments, and scavenging reactive oxygen species (ROS). Together, these biochemical properties may explain the observed improvements in photosynthetic efficiency, nutrient uptake, and overall plant performance under salinity stress^34^.

Although the biomass was subjected to defatting using hexane extraction, the GC–MS analysis still revealed the presence of fatty acids and their derivatives. This can be attributed to the selective nature of hexane, which primarily removes neutral lipids, while polar and structurally bound lipid fractions (e.g., phospholipids and membrane-associated fatty acids) remain in the residual biomass and can be subsequently extracted using ethanol. Therefore, the detected fatty acids likely represent residual or bound forms rather than storage lipids. Importantly, their presence does not contradict the defatting process, but rather highlights the retained bioactive potential of the biomass. These compounds, together with other metabolites identified in the extract, may act synergistically to enhance plant growth and stress tolerance, supporting the concept of valorizing defatted microalgal biomass as an effective biostimulant.

### Impact of salinity stress on barley growth and photosynthetic efficiency

Salinity stress significantly influenced barley growth, as evidenced by the decline in grain weight and electron transport rate (ETR) across the salinity treatments (Table 2). These findings are consistent with previous studies reporting reductions in growth parameters and photosynthetic performance in crops exposed to salinity stress [35]. The reduction in grain weight and ETR under moderate (S1) and high (S2) salinity conditions clearly reflects the detrimental effects of salinity on plant development. High salinity induces osmotic stress, which limits water uptake and turgor maintenance, leading to reduced stomatal conductance, CO₂ assimilation, and overall photosynthetic efficiency. Prolonged exposure further results in ion toxicity, as excessive Na⁺ and Cl⁻ accumulation disrupts K⁺ and Ca²⁺ balance, impairs chloroplast integrity, and inhibits enzyme activity^36^. These combined osmotic and ionic effects ultimately restrict carbon fixation and grain filling.

The observed variation in salinity tolerance among barley genotypes, reflected in grain weight and ETR, aligns with genotype-specific differences in stress responses reported previously^36,37^. Among the tested cultivars, Giza 123 exhibited the highest grain weight and ETR, suggesting a greater capacity for maintaining photosynthetic performance and ion homeostasis under saline conditions^38^. Such genotype-based variability in tolerance likely arises from differences in osmotic adjustment capacity, ion compartmentalization, and the regulation of stress-responsive genes^39^.

### Effect of algal extract on growth and photosynthetic parameters

The application of defatted *Chlorella* sp. biomass, particularly through the combined soaking and foliar spray treatments, led to significant enhancements in grain weight and electron transport rate (ETR) under salinity stress. Similar positive effects of algal extracts on plant growth and photosynthetic activity under saline conditions have been documented in various crops^40^. The bioactive constituents of *Chlorella* sp., including fatty acids and other phytochemicals, are known to stimulate plant growth and mitigate the adverse effects of abiotic stresses such as salinity^29,41]^. In the present study, the seed-soaking treatment exhibited the greatest increases in grain weight and ETR, outperforming foliar spraying, likely due to more efficient absorption of bioactive compounds through root uptake. The observed increase in maximum fluorescence (Fm) further indicates improved photochemical efficiency of photosystem II (PSII), suggesting enhanced photochemical quenching and reduced non-photochemical energy dissipation (NPQ). Such improvements reflect a more efficient utilization of absorbed light energy under saline conditions. Moreover, the significant genotype × treatment interaction underscores the necessity of tailoring biostimulant strategies to specific genotypes. Among the tested cultivars, Giza 123 showed the strongest response, indicating its potential suitability as a target genotype for enhancing salt tolerance through algal-based biostimulant applications aimed at improving barley performance under salinity stress.

### Photosynthetic performance under salinity stress

Interestingly, while salinity reduced biomass and yield, it did not significantly affect quantum yield, diffusion rate, transpiration, or net CO₂ assimilation. This contrasts with several studies reporting salinity-induced declines in photosynthetic parameters^27,29^. One possible explanation is that the salinity levels applied here, although sufficient to reduce yield, were not severe enough to impair core photosynthetic processes in barley. Alternatively, barley may possess physiological mechanisms that help maintain photosynthetic efficiency under mild to moderate salinity stress^27,34^.

In contrast, algal extract application influenced quantum yield, diffusion rate, and net CO₂ assimilation, the soaking treatment providing the highest improvements. This indicates that algal application may enhance photosynthetic efficiency in barley by improving key physiological processes involved in carbon fixation. Previous studies have shown that algal extracts can enhance chloroplast activity, improve stomatal conductance, and increase the efficiency of photosystem II, all of which contribute to better photosynthetic performance^13,35,36^. The results from this study suggest that the beneficial effects of algal treatments on photosynthesis may be partly due to the improvement of carbon assimilation and diffusion rates, which are crucial for plant productivity under stress conditions.

### Chlorophyll fluorescence and photosystem efficiency

The analysis of chlorophyll fluorescence parameters revealed that salinity stress did not significantly alter the initial CO₂ concentration, minimum fluorescence (F₀), maximum fluorescence (Fm), or the Fv/Fm ratio. These findings suggest that salinity had a limited impact on the basic functioning of the photosynthetic apparatus in barley under the experimental conditions. Algal application, especially the soaking treatment, had a significant positive impact on chlorophyll fluorescence parameters. The increased Fm and Fv/Fm ratio observed in the algal-treated plants suggests that algal biostimulants can enhance photosystem II efficiency, thereby improving light capture and energy conversion during photosynthesis. This aligns with previous reports showing that algal treatments can improve chlorophyll fluorescence and photosystem efficiency in crops under stress^36–38^.

### Macronutrient content in barley shoots and grains

Salinity stress induced significant changes in the macronutrient content in barley shoots, with a marked decrease in phosphorus and potassium content and an increase in sodium accumulation under higher salinity levels. This suggests that salinity causes nutrient imbalances in plants, with the accumulation of toxic ions like sodium and the depletion of essential macronutrients like phosphorus and potassium^26,39^. The observed genotype-specific variation in macronutrient content further highlights the importance of selecting salinity-tolerant genotypes that can maintain better nutrient uptake and assimilation under stress conditions. Application of the algal extract markedly enhanced the macronutrient content in barley shoots, particularly increasing phosphorus, potassium, and nitrogen levels. The soaking treatment resulted in the highest nutrient content, indicating that algal extracts can enhance the uptake and assimilation of essential nutrients, potentially mitigating the nutrient imbalances caused by salinity. This finding is consistent with previous studies that have reported improved nutrient uptake and efficiency in plants treated with algal biostimulants^14,18,19^.

In conclusion, the application of algal extract significantly enhanced barley performance under salinity stress, improving both growth and nutrient uptake. Among the tested treatments, the combined seed soaking and foliar spraying method produced the most pronounced improvements, increasing grain weight by approximately 13% compared with foliar spraying alone and by 9% compared with soaking. Individually, soaking and spraying enhanced grain weight by 6–8% relative to the control. This suggests that seed soaking promotes early metabolic activation and osmotic adjustment, whereas foliar spraying supports continued nutrient assimilation and photosynthetic performance during later growth stages. On the other hand, The application of defatted *Chlorella sp.* biomass notably improved the nutritional quality of barley grains by increasing protein content and enhancing photosynthetic pigments such as chlorophylls and carotenoids. These biochemical improvements are particularly valuable under salinity stress, as they not only reflect better metabolic activity but also contribute to enhanced nutritional value of the harvested grains. Higher protein content improves the dietary quality of barley, which is essential for food and feed security in saline-affected regions. Similarly, increased pigment antioxidants such as chlorophylls and carotenoids enhance the plant’s oxidative stress defense mechanisms, contributing to overall stress resilience and potentially sustainable crop performance under adverse environmental conditions. Although the hydroponic system used in this study provided a controlled environment to precisely assess the physiological responses of barley to salinity and algal treatments, it does not fully replicate the complex soil conditions encountered under field cultivation. Factors such as soil texture, microbial interactions, and fluctuating environmental conditions may influence the bioavailability and efficacy of algal biostimulants. Therefore, further multi-season and field-based experiments are needed to validate these findings under natural conditions and to evaluate the scalability and agronomic feasibility of using defatted *Chlorella sp.* biomass in sustainable crop management practices.

## Conclusion

This study demonstrates that lipid-extracted (defatted) Chlorella sp. biomass can serve as a promising biostimulant for barley cultivated under salinity stress, improving growth performance, photosynthetic efficiency, and mineral nutrient status. Among the tested application strategies, seed soaking consistently produced the most pronounced benefits, particularly in the salt-tolerant genotype Giza 123, highlighting the importance of genotype-dependent responsiveness to algal-derived biostimulants. Overall, the results suggest that Chlorella-based biostimulants may represent a sustainable and potentially cost-effective approach to support crop productivity in salt-affected environments. Nevertheless, since the present work was conducted under controlled hydroponic conditions and evaluated a limited number of genotypes, further multi-season and field-scale investigations are required to validate the reproducibility and agronomic feasibility of these findings under diverse environmental and management conditions.

## Electronic supplementary material

Below is the link to the electronic supplementary material.

Supplementary Material 1

## Data Availability

The data supporting the findings of this study are presented in this published article.
