# Supplementary material for: Defatted biomass of the green microalga Chlorella sp. as a sustainable biostimulant to enhance barley growth under saline conditions
Source: Sci Rep. 2026 May 13;16:15064. doi: 10.1038/s41598-026-49609-6 (PMC13171915; doi:10.1038/s41598-026-49609-6)
Supplement: Supplementary file 1 — Supplementary Material 1 [file 41598_2026_49609_MOESM1_ESM.docx]

**Supplementary Table S1. Composition of Hoagland’s Nutrient Solution Used in the Hydroponic Experiment**

| **Component** | **Chemical Form** | **Concentration (mM)** | **Function** |
| --- | --- | --- | --- |
| **Macronutrients** |  |  |  |
| Nitrogen (N) | KNO₃ | 5.00 | Major source of N and K |
| Phosphorus (P) | KH₂PO₄ | 1.00 | Source of P and K |
| Potassium (K) | KNO₃ + KH₂PO₄ | 6.00 | Essential for enzyme activation |
| Calcium (Ca) | Ca(NO₃)₂·4H₂O | 5.00 | Cell wall stability and signaling |
| Magnesium (Mg) | MgSO₄·7H₂O | 2.00 | Chlorophyll component |
| Sulfur (S) | MgSO₄·7H₂O | 2.00 | Component of amino acids |
| **Micronutrients** |  |  |  |
| Iron (Fe) | Fe-EDTA | 0.10 | Electron transport and chlorophyll synthesis |
| Manganese (Mn) | MnCl₂·4H₂O | 0.005 | Cofactor in photosynthetic reactions |
| Zinc (Zn) | ZnSO₄·7H₂O | 0.005 | Enzyme component |
| Copper (Cu) | CuSO₄·5H₂O | 0.001 | Enzyme cofactor |
| Boron (B) | H₃BO₃ | 0.025 | Cell wall formation and membrane integrity |
| Molybdenum (Mo) | (NH₄)₆Mo₇O₂₄·4H₂O | 0.001 | Nitrate reduction |

**Notes**

- The solution was adjusted to **pH 6.0 ± 0.1** before use.
- Renewed every 5 days to maintain ionic balance and prevent nutrient depletion.
- Salinity treatments were established by supplementing with **NaCl and CaCl₂ (1:1 molar ratio)** to reach the desired EC levels (2, 8, and 12 mS/cm)

**Supplementary Table S2.** Previously reported occurrences of bioactive compounds identified in the ethanol extract of *Chlorella sp.* biomass based on GC–MS analysis.

| No | Compound Name | CAS | Formula | MW | RT | Area (%) | Reference(s) |
| --- | --- | --- | --- | --- | --- | --- | --- |
| 1 | ETHYL (9Z,12Z)-9,12-OCTADECADIENOATE | 544-35-4 | C20H36O2 | 308 | 23.54 | 18.72 | Mahmoud et al., 2025; Macías-de la Rosa et al., 2024 |
| 2 | Carbonic acid, eicosyl vinyl ester |  | C23H44O3 | 368 | 15.64 | 13.20 | Macías-de la Rosa et al., 2024 |
| 3 | Hexadecanoic acid, methyl ester | 112-39-0 | C17H34O2 | 270 | 19.60 | 6.10 | Mahmoud et al., 2025; Macías-de la Rosa et al., 2024 |
| 4 | trans-13-Octadecenoic acid, methyl ester |  | C19H36O2 | 296 | 22.37 | 5.70 | Mahmoud et al., 2025; Macías-de la Rosa et al., 2024; Youssef et al., 2021 |
| 5 | 1-Eicosanol | 629-96-9 | C20H42O | 298 | 17.31 | 10.92 | Macías-de la Rosa et al., 2024 |
| 6 | Oleic Acid | 112-80-1 | C18H34O2 | 282 | 23.06 | 8.66 | Mahmoud et al., 2025; Macías-de la Rosa et al., 2024 |
| 7 | Methyl(Z)-5,11,14,17-eicosatetraenoate | 59149-01-8 | C21H34O2 | 318 | 24.59 | 4.47 | Mahmoud et al., 2025; Macías-de la Rosa et al., 2024; El-Hadad et al., 2021 |
| 8 | 1-Hexadecanol | 36653-82-4 | C16H34O | 242 | 13.64 | 6.56 | Mahmoud et al., 2025; El-Saadawi et al., 2022 |
| 9 | 9-OCTADECENOIC ACID (Z) | 112-80-1 | C18H34O2 | 282 | 25.30 | 4.07 | Mahmoud et al., 2025; Macías-de la Rosa et al., 2024 |
| 10 | Methyl stearate | 112-61-8 | C19H38O2 | 298 | 22.78 | 2.95 | Mahmoud et al., 2025; Macías-de la Rosa et al., 2024 |
| 11 | n-Hexadecanoic acid | 57-10-3 | C16H32O2 | 256 | 20.30 | 2.87 | Mahmoud et al., 2025; Macías-de la Rosa et al., 2024 |
| 12 | (Z)-Methyl hexadec-11-enoate | 822-05-9 | C17H32O2 | 268 | 19.24 | 2.23 | Mahmoud et al., 2025 |
| 13 | 6,9,12,15-Docosatetraenoic acid, methyl ester | 17364-34-0 | C23H38O2 | 346 | 22.07 | 1.73 | Mahmoud et al., 2025; El-Hadad et al., 2021 |
| 14 | Neophytadiene | 504-96-1 | C20H38 | 278 | 18.12 | 2.45 | Macías-de la Rosa et al., 2024, El-Hadad et al., 2021 |
| 15 | 3-(N,N-Dimethyllaurylammonio) propanesulfonate | 14933-08-5 | C17H37NO3S | 335 | 12.01 | 1.37 | El-Hadad et al., 2021 |
| 16 | 2,6,10-Dodecatrien-1-ol,3,7,11-trimethyl- | 4602-84-0 | C15H26O | 222 | 24.17 | 1.33 | El-Hadad et al., 2021 |
| 17 | á-Sitosterol | 83-46-5 | C29H50O | 414 | 30.17 | 2.18 | Macías-de la Rosa et al., 2024; El-Hadad et al., 2021 |
| 18 | 1-DOCOSANOL | 661-19-8 | C22H46O | 326 | 26.60 | 0.98 | Macías-de la Rosa et al., 2024; El-Hadad et al., 2021 |
| 19 | HI-OLEIC SAFFLOWER OIL | 8001-23-8 | C21H22O11 | 450 | 27.36 | 0.92 | El-Hadad et al., 2021 |
| 20 | 3,7,11,15-Tetramethyl-2-hexadecen-1-ol | 102608-53-7 | C20H40O | 296 | 18.55 | 0.73 | El-Hadad et al., 2021 |
| 21 | 2-OCTANONE | 111-13-7 | C8H16O | 128 | 15.82 | 0.58 | Macías-de la Rosa et al., 2024; El-Hadad et al., 2021 |
| 22 | 17-Octadecynoic acid | 34450-18-5 | C18H32O2 | 280 | 20.83 | 1.11 | Macías-de la Rosa et al., 2024; El-Hadad et al., 2021 |
| 23 | ISOCHIAPIN B |  | C19H22O6 | 346 | 28.64 | 0.57 | El-Hadad et al., 2021 |
| 24 | Caryophyllene | 87-44-5 | C15H24 | 204 | 10.29 | 0.55 | Macías-de la Rosa et al., 2024; El-Hadad et al., 2021 |
| 25 | [1,1'-Bicyclopropyl]-2-octanoic acid, 2'-hexyl-, methyl ester | 56687-68-4 | C21H38O2 | 322 | 18.22 | 0.37 | El-Hadad et al., 2021 |
| 26 | Methyl tetradecanoate | 124-10-7 | C15H30O2 | 242 | 16.12 | 0.49 | Mahmoud et al., 2025; El-Hadad et al., 2021 |
| 27 | Hexadecanoic acid, 14-methyl-,methyl ester | 2490-49-5 | C18H36O2 | 284 | 21.22 | 0.40 | Macías-de la Rosa et al., 2024; El-Hadad et al., 2021 |
| 28 | 1-CHLOROOCTADECANE | 3386-33-2 | C18H37Cl | 288 | 13.78 | 0.38 | El-Hadad et al., 2021 |

- Mahmoud, M.K., et al., “Edaphic algae as sustainable biofactories for high-valued products,” Egypt. J. Exp. Biol. (Bot.), 2025.
- Macías-de la Rosa, A., López-Rosales, L., Contreras-Gómez, A., Sánchez-Mirón, A., García-Camacho, F., & Cerón-García, M. D. C. (2024). Salinity as an abiotic stressor for eliciting bioactive compounds in marine microalgae. *Toxins*, *16*(10), 425.‏
- Youssef, A.A., et al., “An Evaluation Activity of Some Algal Extracts Against Culex,” Egyptian Journal of Aquatic Biology & Fisheries, 2021.
- El-Hadad, S., et al., “Identification and characterization of bioactive compounds in two algae Chlorophyta species,” Int. J. Ichthyol., 2021.
